# Geographic heterogeneity of the epidemiological impact of the COVID-19 pandemic in Italy using a socioeconomic proxy-based classification of the national territory

**DOI:** 10.3389/fpubh.2023.1143189

**Published:** 2023-04-21

**Authors:** Alessio Petrelli, Martina Ventura, Anteo Di Napoli, Alberto Mateo-Urdiales, Patrizio Pezzotti, Massimo Fabiani

**Affiliations:** ^1^Epidemiology Unit, National Institute for Health, Migration and Poverty (INMP), Rome, Italy; ^2^Unit of Epidemiology, Biostatistics and Mathematical Modelling, Department of Infectious Diseases, Istituto Superiore di Sanità, Rome, Italy

**Keywords:** COVID-19, geographic inequalities, rural, metropolitan, social vulnerabilities

## Abstract

**Objectives:**

This study aimed to evaluate the differences in incidence, non-intensive care unit (non-ICU) and intensive care unit (ICU) hospital admissions, and COVID-19-related mortality between the “inner areas” of Italy and its metropolitan areas.

**Study design:**

Retrospective population-based study conducted from the beginning of the pandemic in Italy (20 February 2020) to 31 March 2022.

**Methods:**

The municipalities of Italy were classified into metropolitan areas, peri-urban/intermediate areas and “inner areas” (peripheral/ultra-peripheral). The exposure variable was residence in an “inner area” of Italy. Incidence of diagnosis of SARS-CoV-2 infection, non-ICU and ICU hospital admissions and death within 30 days from diagnosis were the outcomes of the study. COVID-19 vaccination access was also evaluated. Crude and age-standardized rates were calculated for all the study outcomes. The association between the type of area of residence and each outcome under study was evaluated by calculating the ratios between the standardized rates. All the analyses were stratified by period of observation (original Wuhan strain, Alpha variant, Delta variant, Omicron variant).

**Results:**

Incidence and non-ICUs admissions rates were lower in “inner areas.” ICU admission and mortality rates were much lower in “inner areas” in the early phases of the pandemic, but this protection progressively diminished, with a slight excess risk observed in the “inner areas” during the Omicron period. The greater vaccination coverage in metropolitan areas may explain this trend.

**Conclusion:**

Prioritizing healthcare planning through the strengthening of the primary prevention policies in the peripheral areas of Italy is fundamental to guarantee health equity policies.

## Introduction

Italy has been one of the countries hit hardest by the COVID-19 pandemic. Official estimates report that at the beginning of October 2022, about 41% of the resident population of Italy had contracted the virus (1). This proportion is obviously underestimated, especially since the spread of the Omicron variant, both because only some of the asymptomatic cases have been diagnosed, given that contact tracing has been essentially suspended, and because of the widespread use of COVID-19 home testing kits. By the same date in early October, COVID-19 had caused 177,000 deaths in Italy, with one of the highest mortality rates in the world before the introduction of the vaccine, and with mortality remaining high despite extensive vaccination coverage (1). COVID-19 has contributed to widening socioeconomic inequalities both directly and indirectly. Directly, the most disadvantaged social groups of the population (2, 3), including immigrants (4), have been more seriously affected, in terms of the number of infections and outcomes. With the obvious exception of healthcare professionals, these same disadvantaged individuals have been indirectly impacted by the suspension and rescheduling of all non-urgent care so as to provide medical assistance to COVID-19 patients. During the pandemic, a reduction in access to healthcare services has been observed, in part due to the saturation of availability of services because of the COVID-19 emergency but also due to the perception, real or otherwise, that healthcare facilities are potential sources of infection. The reduction in access has been higher among the most socioeconomically disadvantaged groups of the population (5).

Even before the pandemic, individual socioeconomic inequalities in Italy were compounded by geographic area-related inequalities. Regardless of socioeconomic level, in fact, the life expectancy of individuals residing in the southern regions of Italy is 1 year less than that of persons residing in the central and northern regions (6). This is true for education level as well, which is known to be a robust proxy for individual socioeconomic level (6). The differences in life expectancy between the North and the South, which decreased in 2020 as an effect of the pandemic (the first wave struck especially the northern regions), increased in 2021, reaching a value of 1.7 years (7).

A considerable amount of literature has been published on the evaluation of the role of living environment in health behaviors and outcomes, analyzing the geographic heterogeneity in regional areas that do not necessarily correspond to regional, provincial, or municipal administrative areas. This literature has focused in particular on the concepts of urbanization and of population density (8).

Living in an urban context is associated with a higher incidence of environmental pollution-related diseases (9), while in rural areas higher incidences of and mortality due to diabetes (8, 10, 11), screening-preventable (12, 13) or lifestyle-associated (13) cancer, and suicide (14) have been observed. Regarding cardiovascular diseases, the evidence has not always been consistent (8, 15). The differing distribution of distal social determinants such as poverty, low socioeconomic level (16), and/or belonging to an ethnic minority (17), associated with an unhealthy lifestyle, are often at the root of the geographic differences observed.

The COVID-19 pandemic has had a very heterogenous impact on populations. Known risk factors like population density of the area of residence, dwelling density, and mobility (18) have interacted with conditions of health and social vulnerability, determining worse outcomes in disadvantaged groups. The term syndemic has often been used to describe a health outcome determined by indissolubly intersecting diseases and social factors (19, 20).

In this sense, although an urban setting is a potential risk factor for infection, it has been seen that the pandemic has struck rural areas in a number of countries (21–24) more harshly in terms of the number of infections and of mortality, except in the first wave.

Nevertheless, studies on geographic differences in the epidemiological impact of the pandemic have been mainly conducted in the United States, especially, and some developing countries (19, 20, 25–29), despite the topic being extremely relevant to public health decision-making worldwide, including Italy (30). In Italy, the National Strategy of “Inner Areas” (SNAI) recently created a new classification of municipalities, with six categories on the basis of the distance to the nearest metropolitan area: metropolitan (municipal or intermunicipal), peri-urban, intermediate, peripheral, ultra-peripheral. The “inner areas” include peripheral and ultra-peripheral municipalities and are characterized by the paucity of essential services such as education, mobility, and healthcare. These areas thus have a high risk of social deprivation and health and social care vulnerability, factors that are closely correlated with COVID-19.

## Objective

The aim of the study was to evaluate the differences in incidence, non-intensive care units (non-ICU) and intensive care unit (ICU) admissions, and mortality between the “inner areas” and the metropolitan areas of Italy.

## Methods

### Study design

The study was based on the resident population in Italy on 1 January 2020. The database used to quantify the cases and outcomes of COVID-19 was the COVID-19 Integrated Surveillance System of the Italian National Institute of Health (24). In accordance with Italian law N. 52 of 19 May 2022, following the law decree N. 24 of 24 March 2022 (Article n. 13), the information on vaccination coverage was retrieved by the Italian National Institute of Health using data from the National Immunisation Information System of the Italian Ministry of Health.

### Exposure

The exposure variable for the study was residence in an “inner area” of Italy, as defined based on the municipality.

To classify the municipalities of Italy, we adopted the concept of “inner areas” according to the meaning and the methodology defined in the 2014–2020 SNAI planning cycle, updated for the 2021–2027 planning cycle. The SNAI is a strategic plan of the Italian Territorial Cohesion Agency,^1^ a public body supervised by the President of the Council of Ministers whose objective is to promote economic development and territorial cohesion so as to eliminate the territorial differences throughout the country and to strengthen the administrative abilities of the administrations.

The general objective is to support and develop rural areas that are in decline or at demographic risk but whose active community supervision is crucial to the overall maintenance of the territory in terms of hydrogeological profile, landscape, and cultural identity (31). “Inner areas” are often characterized by considerable environmental (water resources, agricultural systems, forests, natural and human landscapes) and cultural (archaeological sites, historical settlements, abbeys, small museums, craft centers) resources. They therefore have great tourism potential, but they are far from the main cities that provide essential services such as education, health, and mobility, all of which are available in metropolitan areas.

The classification adopted defines a metropolitan area (municipal or intermunicipal) as contiguous municipalities or groups of municipalities that can jointly provide the following essential public services: at least one classical or scientific high school (*liceo*) and one vocational school or technical institute, an Urgent Care center, and a train station.

**Figure 1:**
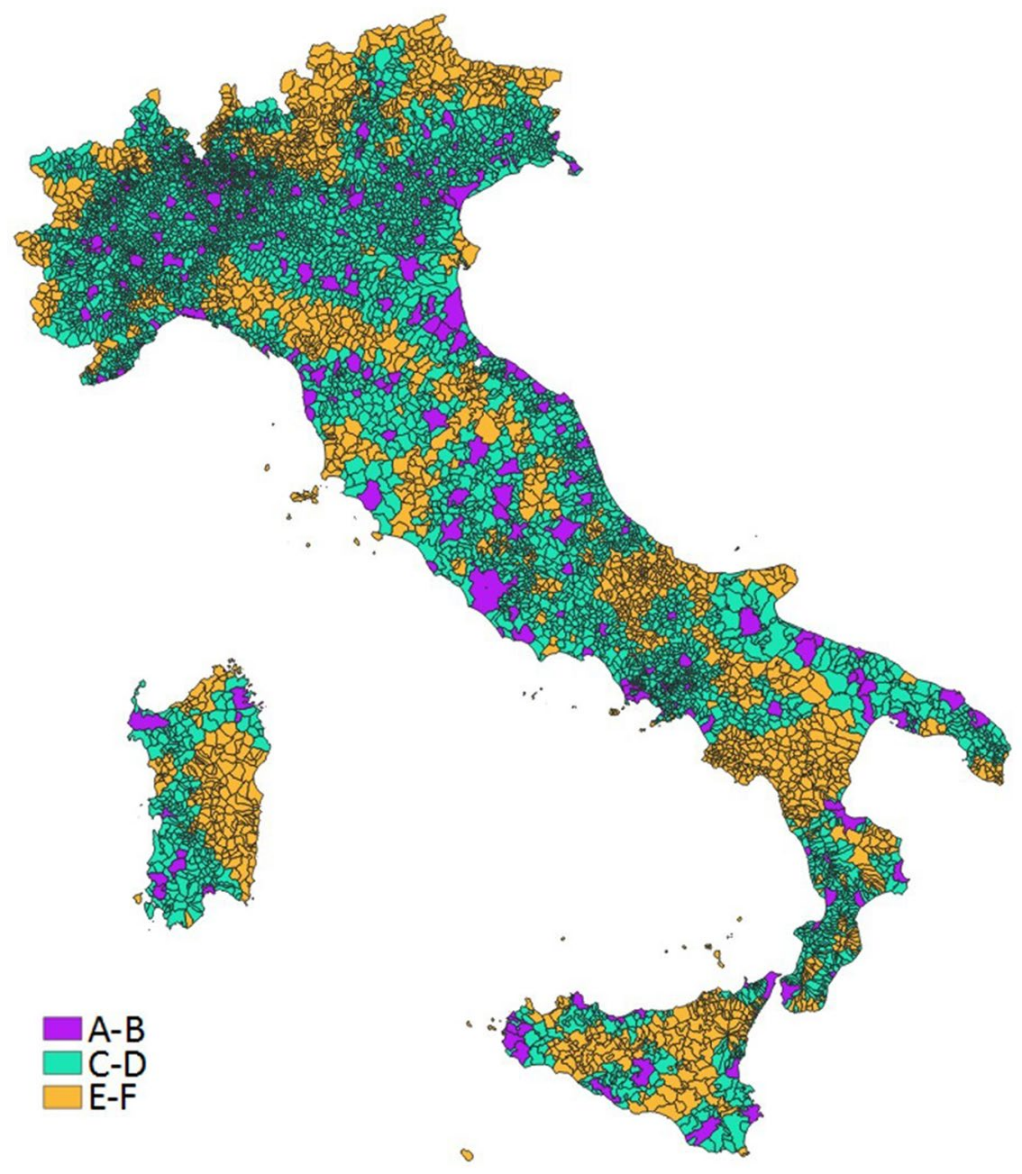
Distribution of the municipalities in metropolitan (A-B), peri-urban/intermediate (C-D) and inner (E-F) areas.

The municipalities that are not part of a metropolitan area (municipal or intermunicipal) are classified in one of four categories (peri-urban, intermediate, peripheral, ultra-peripheral) according to the distance in terms of average driving time to the nearest metropolitan area. “Inner areas” include peripheral and ultra-peripheral categories. The distance is categorized according to the mean, the third quartile, and the 95th percentile of overall distribution. Specifically, the classification of Internal Areas updated for the 2021–2027 planning cycle was used, which refers to all the Italian municipalities in 2020 (*n* = 7,903). The municipalities with a driving time distance from a municipal (A) or intermunicipal (B) metropolitan area closest to the distribution mean value (27.7 min) were classified as peri-urban (C – 3,828 municipalities); over that value and up to the value of the third quartile (40.9 min), they were classified as intermediate (D – 1,928 municipalities). Over that value and up to the 95th percentile (66.9 min), they were classified as peripheral (E – 1,524 municipalities). Finally, those over the 95th percentile (more than 66.9 min) were classified as ultra-peripheral (F – 382 municipalities). For the purpose of the study, the six categories were aggregated into three classes: metropolitan areas (A + B), peri-urban/intermediate areas (C + D), and “inner areas” (peripheral/ultra-peripheral) (E + F). Figure 1 illustrates the distribution of the municipalities according to the adopted classification.

### Study period

The period of time considered in this study was from the beginning of the pandemic in Italy (20 February 2020) to 31 March 2022.

The analyses were stratified in periods based on the predominance of the different variants during the course of the pandemic (32). Specifically, four time periods were defined as follows:

1. 20 February–31 December 2020, characterized primarily by the original Wuhan strain
2. 1 January–30 June 2021, characterized primarily by the Alpha variant
3. 1 July–31 December 2021, characterized primarily by the Delta variant
4. 1 January–31 March 2022, characterized primarily by the Omicron variant

### Outcomes

The following outcomes were analyzed:

- Incidence of diagnosis of SARS-CoV-2 infection confirmed by PCR or, from 15 January 2021, by antigen test;
- Incidence of non-ICU hospital admissions, ICU hospital admissions, and death within 30 days from diagnosis.

COVID-19 vaccination access was also evaluated.

### Statistical analyses

The demographic and territorial characteristics of the resident population of each of the three classes of municipalities (metropolitan areas, peri-urban/intermediate areas, inner areas) are described according to the following variables: population density (people per km^2^), surface area, percentage of population in the municipalities with <10,000 inhabitants and more than 50,000 inhabitants, sex, age class (0–14, 15–29, 30–44, 45–59, 60–74, and >74 years), geographic area of residence (North-West, North-East, Center, and South and Islands), *per capita* taxable income (measured in Euro) and social and material vulnerability index. Information about the level of social and material vulnerability of the municipality of residence was retrieved from the 8milaCensus platform managed by Istat (33). This multidimensional indicator, updated to 2011, has been computed by ISTAT at the municipality level, on the basis of seven socio-economic indicators measuring the incidence of: population with age between 25 and 64 that is illiterate or without qualification; families with at least six members; single parent families (with age of parent up to 64) over the total of families; families with possible welfare poverty; population living in severely crowded conditions; young people (15–29 years) without occupation; families with children with potential economic poverty.

The distributions of COVID-19 cases, non-ICU and ICU hospital admissions, and deaths were also analyzed.

Crude and standardized rates by age, with 95% confidence intervals (CI), were calculated for all the study outcomes. Standardization by age was performed with the direct standardization method, taking as the standard population of reference that of the total resident population on 1 January 2020. The association between the type of area of residence and each outcome under study was evaluated by calculating the ratios between the standardized rates (RR) with 95% CI, considering the AB municipality class as the reference category. All the analyses were stratified by period of observation. The analysis further stratified by geographic area of residence is provided in the Supplementary material.

A supplementary analysis was performed on COVID-19 vaccination access. For the population of individuals at least 5 years of age, those who had received at least one dose of the vaccine during the period under study were considered vaccinated, and the crude and standardized rates of vaccination were calculated by age (with relative 95% CI), stratified by type of municipal area and of geographic area of residence. Any difference between areas in terms of vaccination access were evaluate using the calculation of the RR with 95% CI.

The analyses were performed using the SAS 9.3 software.

## Results

According to the criteria of the classification adopted in this study, the “inner areas” of Italy cover 33.7% of the total national surface area and have 9.1% of the population, which in 2020 was 59,641,488 inhabitants. Of this total number of residents in the “inner areas,” 63.4% live in the South and Islands, mainly in municipalities with a population of fewer than 10,000 inhabitants (63.6%) or with a very low population density of 53.6 people per km^2^, against the 789 people per km^2^ in metropolitan areas. In all the geographic areas the average pro capita taxable income was higher in the metropolitan areas, decreasing in the peri-urban/intermediate and again in the inner areas, which had the lowest values. A north–south gradient was also observed for all types of areas. Concerning the proportion of the population living in a condition of potentially serious social and material vulnerability, the lowest values were observed in the North of Italy (2.4% and 4.2%, in the inner areas of the North-West and the North-East, respectively). Significantly higher proportions were found in the Center (26.5% in the inner areas) and especially in the South and Islands (84.4% in the metropolitan areas; Table 1).

**Table 1:** Demographic and territorial characteristics of the resident population of metropolitan areas, peri-urban/intermediate and inner areas.

	Type of area						Total
	Metropolitan		Peri-urban/intermediate		Inner		
Population density (people per km^2^)	789		185.6		53.6		197.4
Surface area (%)	9.3		57		33.7		100
% population resident in municipalities > 50,000 inhabitants	84.1		5.6		1.3		34.5
% population resident in municipalities < 10,000 inhabitants	0.3		45.8		63.6		30.5
Population	**N**	**Row %**	**N**	**Row %**	**N**	**Row %**	**N**
Total	22,235,272	37.3	31,959,035	53.6	5,447,181	9.1	59,641,488
Sex	**N**	**Col %**	**N**	**Col %**	**N**	**Col %**	**N**
M	10,656,285	47.9	15,714,537	49.2	2,679,274	49.2	29,050,096
F	11,578,987	52.1	16,244,498	50.8	2,767,907	50.8	30,591,392
Age class	**N**	**Col %**	**N**	**Col %**	**N**	**Col %**	**N**
0–14	2,797,537	12.6	4,272,663	13.4	657,354	12.1	7,727,554
15–29	3,276,155	14.7	4,843,140	15.2	836,143	15.4	8,955,438
30–44	4,102,231	18.4	5,966,309	18.7	973,556	17.9	11,042,096
45–59	5,273,366	23.7	7,627,866	23.9	1,262,728	23.2	14,163,960
60–74	3,979,202	17.9	5,680,507	17.8	1,029,015	18.9	10,688,724
75+	2,806,781	12.6	3,568,550	11.2	688,385	12.6	7,063,716
Geographic area^a^	**N**	**Col %**	**N**	**Col %**	**N**	**Col %**	**N**
North-West	4,275,406	19.2	6,628,128	20.7	724,003	13.3	11,627,537
North-East	5,903,317	26.5	9,612,967	30.1	472,395	8.7	15,988,679
Center	5,862,355	26.4	5,171,097	16.2	797,640	14.6	11,831,092
South and Islands	6,194,194	27.9	10,546,843	33	3,453,143	63.4	20,194,180
Population living in a condition of potential serious social and material vulnerability^b^	**N**	**Col %**	**N**	**Col %**	**N**	**Col %**	**N**
North-West	14,6,959	2.5	155,047	1.6	11,451	2.4	313,457
North-East	28,290	0.7	60,765	0.9	30,525	4.2	119,580
Center	269,857	4.6	1,034,799	20.0	211,175	26.5	1,515,831
South and Islands	5,225,123	84.4	7,074,534	67.1	2,368,782	68.6	14,668,439
Pro capita taxable income (Euro)^a^^,^^c^							
North-West	24,915		21,057		18,046		22,392
North-East	22,767		20,330		19,305		21,152
Center	22,673		18,279		17,605		20,405
South and Islands	18,690		15,052		14,021		15,952

a North-West (Piedmont, Val d’Aosta, Lombardy, Liguria); North-East (Veneto, Trentino Alto Adige, Friuli Venezia Giulia, Emilia Romagna); Center (Tuscany, Umbria, Marche, Lazio); South (Abruzzo, Molise, Campania, Apulia, Basilicata, Calabria) and Islands (Sicily, Sardinia).

b First quartile of the distribution of the social and material vulnerability index.

c Source: http://dati.istat.it.

Table 2 reports the distribution of COVID-19 cases, non-ICU and ICU hospital admissions, deaths, and the crude and standardized rates of the outcomes under study, stratified by period, and class of municipalities. During the study period, a total amount of 14,364,240 cases of COVID-19, 459,249 non-ICU and 63,582 ICU hospital admissions, and 132,874 deaths were observed. In the inner areas, 7.8% of the total number of SARS-CoV-2 infections were registered, 7% of non-ICU admissions, 6.6% of ICU admissions, and 7.4% of deaths.

**Table 2:** Distribution of COVID-19 cases, non-ICU and ICU hospital admissions, and deaths. Crude and age-standardized rates, with 95% confidence intervals (CI) for all the study outcomes, by class of municipalities and time period.

	Total	%	Wuhan				Alpha				Delta				Omicron			
			*N*	Crude rate *1000	Std rate *1000	95%CI	*N*	Crude rate *1000	Std rate *1000	95%CI	*N*	Crude rate *1000	Std rate *1000	95%CI	*N*	Crude rate *1000	Std rate *1000	95%CI
Incidence	14,364,240	100	2,115,782	35.48	35.48	(35.43–35.52)	2,050,899	34.39	34.39	(34.34–34.43)	2,118,525	35.52	35.52	(35.47–35.57)	8,079,034	135.46	135.46	(135.37–135.55)
Metropolitan areas	5,398,467	37.6	814,140	36.61	36.52	(36.44–36.60)	748,313	33.65	33.73	(33.66–33.81)	811,636	36.50	36.78	(36.70–36.86)	3,024,378	136.02	137.00	(136.85–137.16)
Peri-urban/intermediate areas	7,842,018	54.6	1,167,550	36.53	36.66	(36.60–36.73)	1,144,136	35.80	35.73	(35.66–35.79)	1,156,556	36.19	35.94	(35.88–36.01)	4,373,776	136.86	135.84	(135.71–135.96)
Inner areas	1,123,755	7.8	134,092	24.62	24.52	(24.39–24.65)	158,450	29.09	29.24	(29.09–29.38)	150,333	27.60	27.90	(27.76–28.04)	680,880	125.00	127.01	(126.71–127.32)
Non-ICUs hospitalization	459,249	100	188,913	3.17	3.17	(3.15–3.18)	135,146	2.27	2.27	(2.25–2.28)	56,513	0.95	0.95	(0.94–0.96)	78,677	1.32	1.32	(1.31–1.33)
Metropolitan areas	184,037	40.1	75,422	3.39	3.30	(3.28–3.33)	53,454	2.40	2.35	(2.33–2.37)	23,061	1.04	1.02	(1.00–1.03)	32,100	1.44	1.40	(1.38–1.41)
Peri-urban/intermediate areas	243,051	52.9	101,555	3.18	3.27	(3.25–3.29)	72,002	2.25	2.31	(2.29–2.33)	29,258	0.92	0.94	(0.92–0.95)	40,236	1.26	1.30	(1.29–1.31)
Inner areas	32,161	7.0	11,936	2.19	2.10	(2.06–2.14)	9,690	1.78	1.72	(1.68–1.75)	4,194	0.77	0.75	(0.72–0.77)	6,341	1.16	1.11	(1.08–1.14)
ICU hospitalization	63,582	100	29,446	0.49	0.49	(0.48–0.50)	22,689	0.38	0.38	(0.38–0.39)	6,471	0.11	0.11	(0.10–0.11)	4,976	0.08	0.08	(0.08–0.09)
Metropolitan areas	25,327	39.8	12,093	0.54	0.53	(0.52–0.54)	8,863	0.40	0.39	(0.38–0.40)	2,483	0.11	0.11	(0.11–0.11)	1,888	0.08	0.08	(0.08–0.09)
Peri-urban/intermediate areas	34,055	53.6	15,708	0.49	0.50	(0.49–0.51)	12,362	0.39	0.39	(0.39–0.40)	3,419	0.11	0.11	(0.10–0.11)	2,566	0.08	0.08	(0.08–0.09)
Inner areas	4,200	6.6	1,645	0.30	0.29	(0.28–0.31)	1,464	0.27	0.26	(0.25–0.27)	569	0.10	0.10	(0.09–0.11)	522	0.10	0.09	(0.08–0.10)
Mortality	132,874	100	70,885	1.19	1.19	(1.18–1.20)	34,928	0.59	0.59	(0.58–0.59)	10,577	0.18	0.18	(0.17–0.18)	16,484	0.28	0.28	(0.27–0.28)
Metropolitan areas	51,589	38.8	27,178	1.22	1.16	(1.14–1.17)	13,807	0.62	0.59	(0.58–0.60)	4,261	0.19	0.18	(0.18–0.19)	6,343	0.29	0.27	(0.26–0.28)
Peri-urban/intermediate areas	71,455	53.8	39,135	1.22	1.29	(1.28–1.31)	18,487	0.58	0.61	(0.60–0.62)	5,343	0.17	0.18	(0.17–0.18)	8,490	0.27	0.28	(0.28–0.29)
Inner areas	9,830	7.4	4,572	0.84	0.78	(0.76–0.80)	2,634	0.48	0.45	(0.43–0.47)	973	0.18	0.17	(0.16–0.18)	1,651	0.30	0.28	(0.27–0.29)

Crude and age-standardized rates, with 95% confidence intervals (CI) for all the study outcomes, by class of municipalities and time period.

In the first two periods (Wuhan and Alpha), lower rates for all of the outcomes under study were observed in the inner areas compared to those in the metropolitan areas and in the peri-urban/intermediate areas. The incidence and non-ICU admission rates observed in the inner areas remained lower than those in the other areas in the subsequent periods (Delta and Omicron) as well, while no difference between areas was observed for the more serious outcomes (ICU admission and death).

The analysis of the rates stratified by geographic area of residence showed strong geographic heterogeneity (Supplementary Table 1): while the trend of the rates for the Center and for the South and Islands was in line with those at the national level, in the North it was not. Especially in the second period in both the North-East and North-West, the incidence of COVID-19 and non-ICU admissions were higher in the inner areas than in the metropolitan and peri-urban/intermediate areas. Furthermore, rates of ICU admissions (in the North-East in the first and second period) and of mortality (in the first period) were higher in the inner areas than in the metropolitan areas.

**Figure 2:**
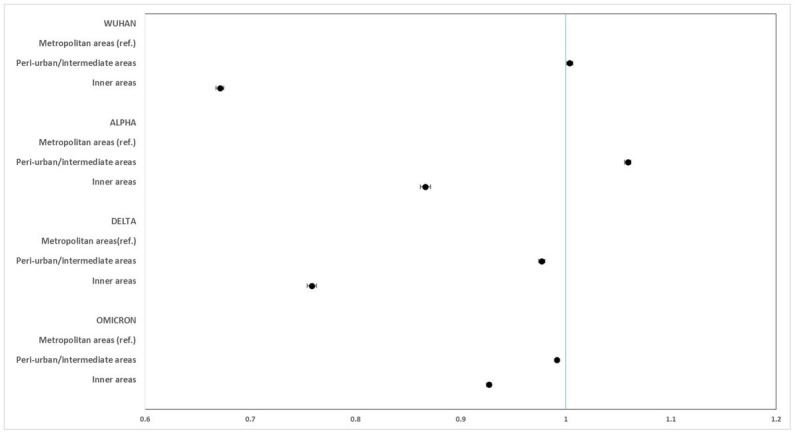
Age-adjusted rate ratios with 95%CI of COVID-19 incidence, by study period and class of municipalities. Metropolitan areas as reference category.

**Figure 3:**
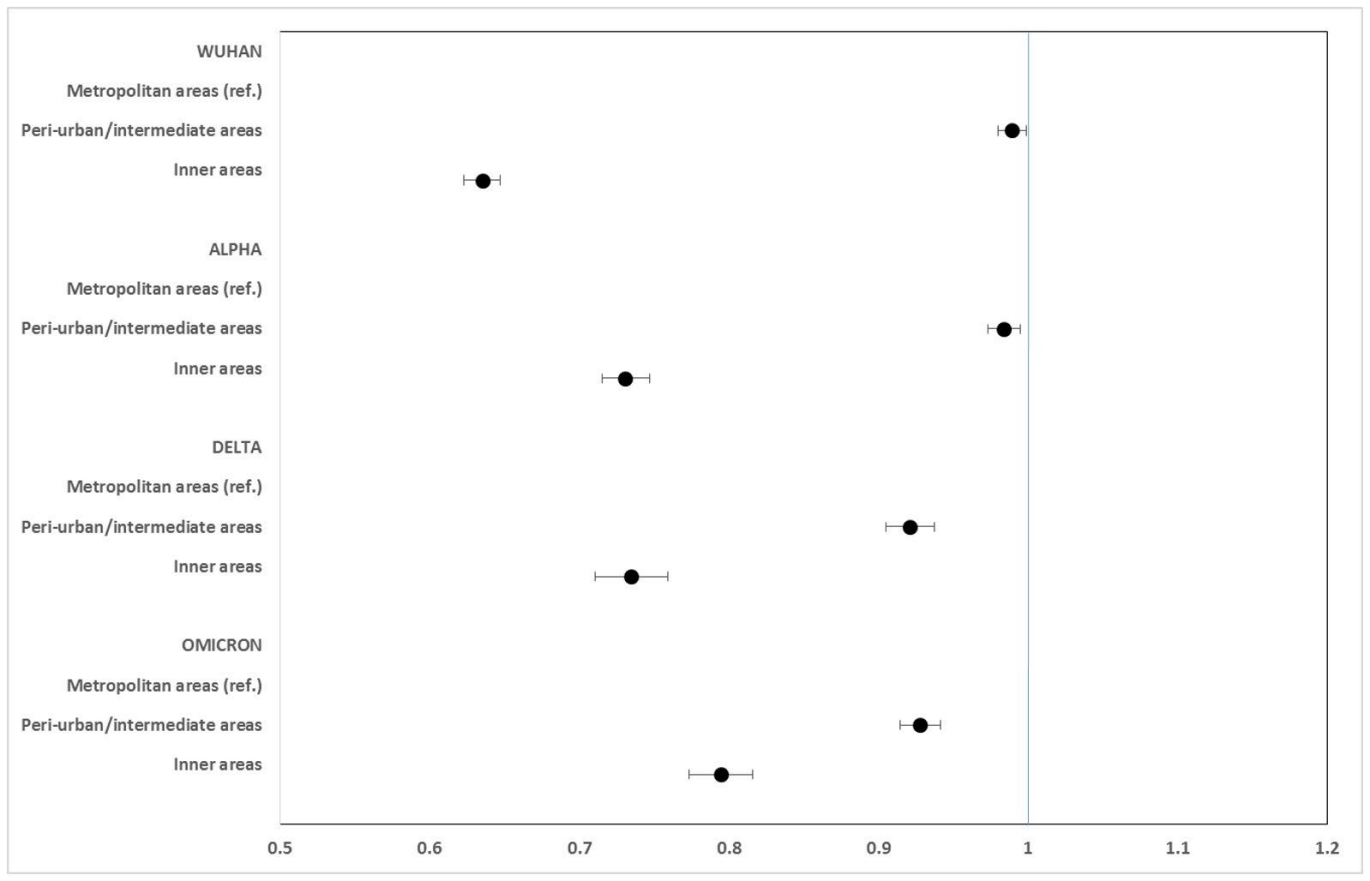
Age-adjusted rate ratios with 95%CI of non-ICU hospitalization, by study period and class of municipalities. Metropolitan areas as reference category.

**Figure 4:**
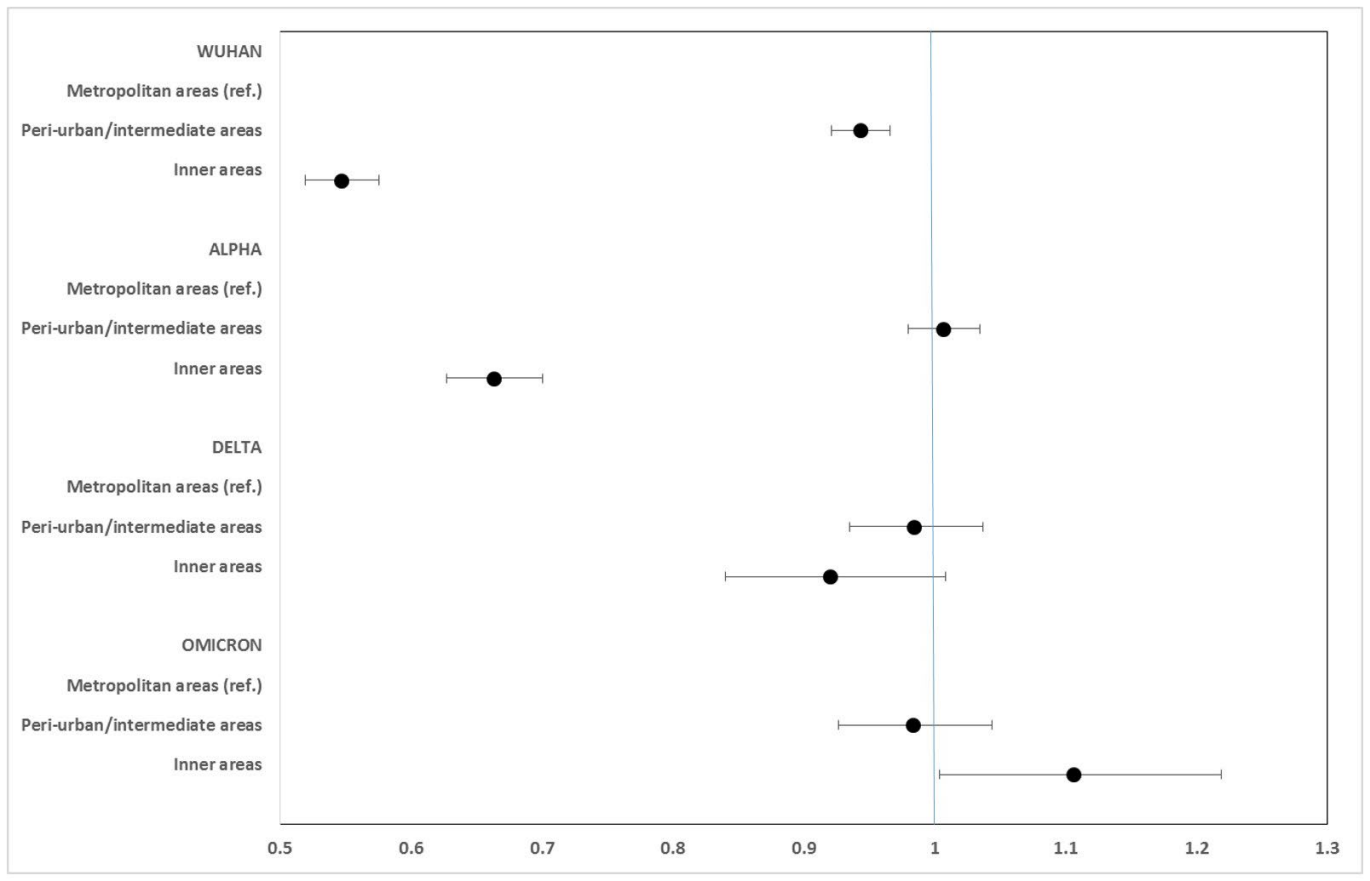
Age-adjusted rate ratios with 95%CI of ICU hospitalization, by study period and class of municipalities. Metropolitan areas as reference category.

**Figure 5:**
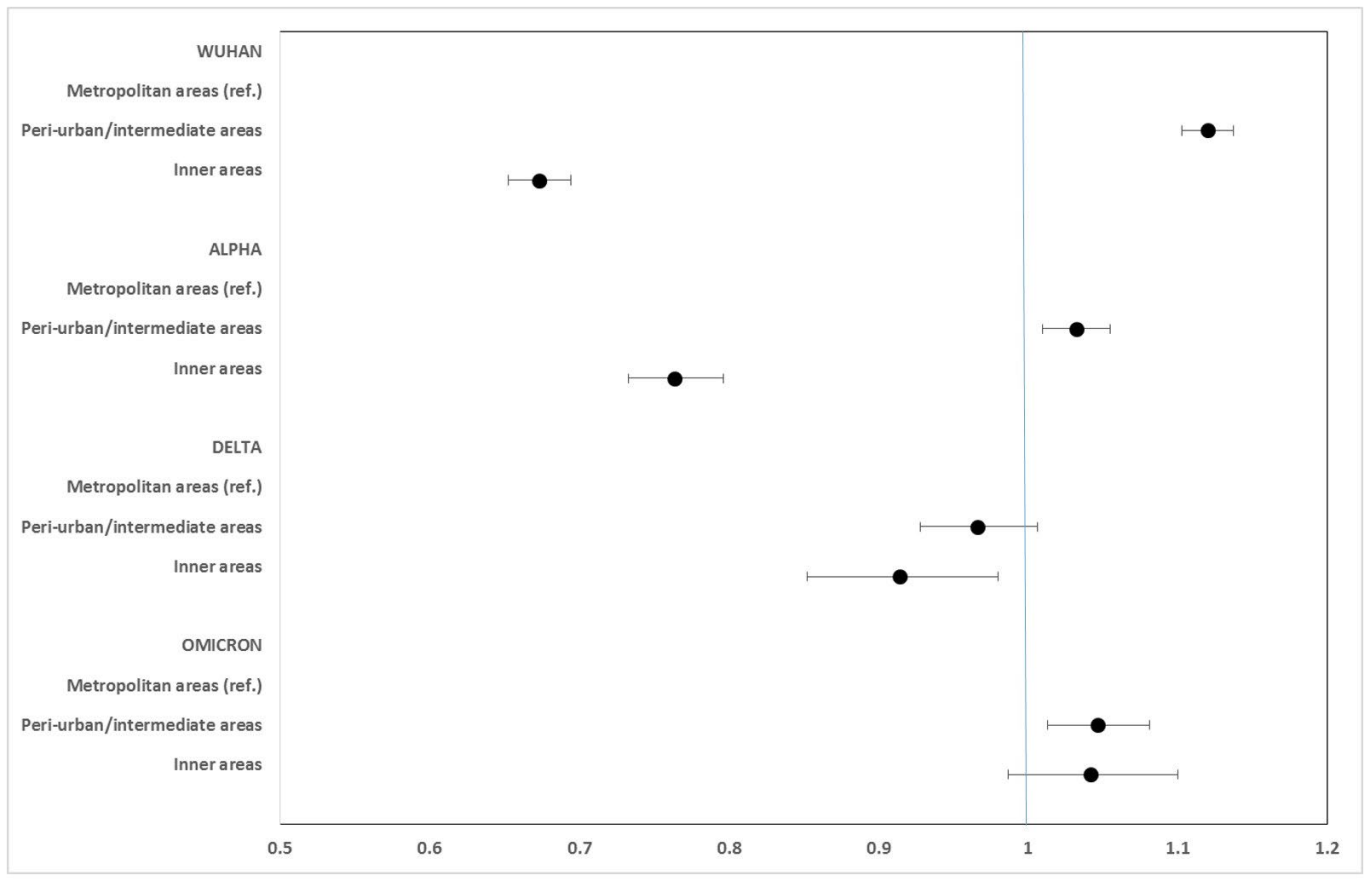
Age-adjusted rate ratios with 95%CI of mortality, by study period and class of municipalities. Metropolitan areas as reference category.

Figures 2–5 report the age-adjusted RR with relative 95% CI, stratified by period and class of municipalities for incidence, non-ICU admission, ICU admission, and death.

Regarding the incidence of COVID-19, rates in the inner areas in all phases of the pandemic were lower than those in the metropolitan areas, particularly in the periods of the original strain (RR: 0.672; 95% CI: 0.667–0.675), while slighter differences were seen during the Omicron variant phase (RR: 0.927; 95% CI: 0.925–0.930).

Non-ICU admission rates were always lower in the inner areas than in the metropolitan areas, especially during the Wuhan + Alpha phase (RR: 0.635; 95% CI: 0.623–0.648) compared to the Omicron phase (RR: 0.795; 95% CI: 0.773–0.816).

ICU admission rates were markedly lower in the inner areas during the Wuhan (RR: 0.547; 95% CI: 0.519–0.576) and Alpha (RR: 0.663; 95% CI: 0.628–0.701) phases. No significant differences were seen during the Delta phase, while a slight excess risk in the inner areas was observed in the Omicron phase (RR: 1.106; 95% CI: 1.004–1.219).

Mortality rates for COVID-19 were considerably lower in the inner areas during the first phase (Wuhan) of the pandemic (RR: 0.673; 95% CI: 0.652–0.695). However, this protection progressively diminished during the Alpha and Delta phases, and a slight excess risk was observed in the Omicron phase, at the limit of statistical significance (RR: 1.042; 95% CI: 0.987–1.100).

The analysis of the data from the National Immunisation Information System showed national-level crude and standardized rates of COVID-19 vaccination access that were lower in the inner areas than in metropolitan areas (77.1 and 85.8%, respectively). The same was seen in all geographic areas, with more marked differences in the South and Islands, where vaccination access was even considerably lower (for all types of areas) than in the other geographic areas (Supplementary Table 2).

## Discussion

This study aimed to evaluate the epidemiological impact of the COVID-19 pandemic on the inner areas of Italy. These areas include rural areas, those that have low or medium population density, and small villages and towns, in line with the methodology used for the classification. These areas are at high risk of social deprivation as they are considerably far from services of education, healthcare, and rail transport, as well has having lower income levels. The results of the study show that the incidence of SARS-CoV-2 infection and non-ICU admissions were lower in the inner areas of the country than in the metropolitan areas in all of the time periods examined. These results are in line with those of another Italian study, which, however, used a different classification of the territory (34). The inner areas have a much lower population density than do the metropolitan areas as well as less mobility; both are important risk factors for the spread of infection, which could at least partially explain why there has been a lower incidence of COVID-19 in these areas. In the United States, instead, where most of the evidence on the geographic differences in the impact of the pandemic comes from, higher incidence rates were seen in rural areas (21, 35), leading to the hypothesis that the differences may depend mainly on a lower perception of risk and on a lower adherence to infection prevention measures there than in urban areas (21, 36).

We observed lower infection and non-ICU admission rates in rural areas in all of the time periods examined. ICU admission and mortality rates were also lower in the inner areas than in metropolitan areas, but only during the periods of the Wuhan strain and the Alpha variant. No differences were observed in the Delta variant period, and there was a higher risk of ICU admission and mortality in the inner areas during the Omicron variant phase.

Furthermore, additional analyses stratified by geographic area (Supplementary Table 1) showed that the national trend was the result of two opposing phenomena: while in the North the highest rates were in the intermediate areas, and in the Omicron phase, also in the inner areas, in the Center and the South rates were decidedly much higher in the metropolitan areas. Thus, one could speculate that living in the inner areas was protective against infection and non-ICU care admission, but that once infected, the probability of worse outcomes was greater, particularly in the most recent periods of the pandemic. While a number of factors may have contributed to determining this scenario, the heterogeneity in vaccination access throughout the country may at least partially explain the phenomenon. As a matter of fact, the analyses performed on the data from the COVID-19 National Vaccination Registry (Supplementary Table 2) show that vaccine coverage with at least two doses was lower in the inner areas than in metropolitan areas in all geographic areas, particularly in the North-West and in the South. Other studies that compared COVID-19 vaccine coverage in urban and rural areas have shown lower vaccination access in rural areas (37, 38). The progressive inversion of the trend seen in the last two observation periods, especially in terms of the most serious clinical outcomes (ICU admission and death), may partially reflect the greater vaccine coverage achieved in metropolitan areas than in the inner areas starting from the second half of 2021, when the vaccination campaign had at that point been extended to almost the entire general population.

Although it is not possible to determine any geographic heterogeneity in terms of the comorbidities of COVID-19 patients, one could hypothesize that there is a greater proportion of subjects vulnerable to the outcomes of COVID-19 in the inner areas; previous analyses have shown higher mortality in inner areas for all causes and for stroke as well as for ischemic heart disease among males (39). Evidence from the U.S. shows a strong interaction between the geographic heterogeneity of the impact of COVID-19 and factors related to social vulnerabilities, such as occupation (25, 40). An Italian study has shown a higher prevalence of overweight and obesity in the rural areas and mountains of Veneto, a large region in the northeast of Italy (41). However, in our study, the worsening of outcomes in the inner areas was seen only in the most recent phases of the pandemic, which seems therefore to limit this possibility. It is plausible, in fact, that factors tied to the syndemic interpretation of the impact of the pandemic and to access to healthcare, such as distance and/or scarcity of healthcare services, would have been apparent from the beginning of the pandemic. In this light, the solidity of the Italian National Health System must be highlighted, especially the hospitals, which are characterized by universal access.

### Strengths and limitations

To our knowledge, this is the first study to evaluate the heterogeneity of the impact of the COVID-19 pandemic on metropolitan and inner areas of Italy. The classification criterion adopted by the National Strategy for the “Internal Areas” developed by the Territorial Cohesion Agency as of 2013 was used to achieve the aim of this study. This multidimensional classification of the territory is based on the education, healthcare, and public transportation services available. As we believed that a purely demographic and/or orographic criterion would not allow an analysis of the complex characteristics of the Italian territory, the classification system of inner areas made it possible to highlight those areas at greater risk of socioeconomic and healthcare vulnerability. The considerable heterogeneity in economic and social development throughout Italy strongly depends on easy access to services, which determine how attractive the population considers an area. Since the end of World War II, many inner areas have undergone an intense process of marginalization because of the scarcity of local services and of employment opportunities, thereby causing migration flows toward large cities. More recently, many of these areas have not exploited opportunities for economic valorization, fundamental to keeping local economies alive and attractive. To this can be added natural events, in particular earthquakes, which have further led to abandoning these areas. The result has been a progressive demographic decrease, a fall in employment, and an additional, progressive reduction in the quality and quantity of public services provided at the local level. This phenomenon has affected the country everywhere.

Nevertheless, the classification system adopted does not allow for a comparison with other studies because it is not validated, as are not most of the indicators of urbanization used in the literature (42). This is therefore a limitation of the study.

Furthermore, because the data available to us are aggregated, not individual-based, it is not possible to evaluate the effect of important determinants of health, for example, the presence of disease, lifestyle, and/or socioeconomic characteristics, the distribution of which throughout the national territory could account for some of the differences observed. Another aspect linked to this limitation is that the geographic categories considered in our study assume a homogenous risk within the areas. However, there may be some communities at greater risk than others, which would better explain geographic differences and would thus contribute to fine tuning targeting interventions.

Finally, we must acknowledge the possibility that the number of diagnoses of infection (especially when asymptomatic) may have been underestimated in the inner areas, especially in the early period of the pandemic, when access to diagnostic tests may have been more difficult in those areas due to the presences of fewer access points.

### Conclusion

Our study highlights that the COVID-19 pandemic has also affected dimensions of health inequality that are not among those usually observed, with outcomes differing among the areas of residence and in the time periods of the pandemic examined. Of particular interest is the observation of a trend toward worse health outcomes in the inner areas as the pandemic has progressed, plausibly due to a lower vaccination coverage compared to that in metropolitan areas. This phenomenon underlines the need to strengthen the vaccination campaign in the inner areas of Italy. More generally, questions must be posed concerning the presence of factors of need, demand, and supply which may determine the differences in health of the populations of “metropolitan” and “inner” areas, more so in consideration of the fact that there is potentially greater vulnerability among the residents of inner areas, with the higher prevalence of chronic diseases such as diabetes and cardiovascular diseases.

Equity in access to healthcare in the inner areas of Italy and the need to strengthen primary prevention policies especially in these areas thus confirm the necessity of prioritizing healthcare planning that is oriented toward health equity.

## Data availability statement

The data analyzed in this study is subject to the following licenses/restrictions: because of data sharing legal restrictions, the dataset including individual records cannot be made publicly available. However, aggregated data will be shared on reasonable request to the corresponding author (AP). Requests to access these datasets should be directed to AP, alessio.petrelli@inmp.it.

## Ethics statement

Ethical review and approval was not required for the study on human participants in accordance with the local legislation and institutional requirements. Written informed consent from the participants’ legal guardian/next of kin was not required to participate in this study in accordance with the national legislation and the institutional requirements.

## Author contributions

AP, AN, MV, AM-U, PP, and MF contributed to conception and design of the study. MV and MF organized the database. MV performed the statistical analysis. AP wrote the first draft of the manuscript. MV wrote sections of the manuscript. All authors contributed to manuscript revision, read, and approved the submitted version.

## Conflict of interest

The authors declare that the research was conducted in the absence of any commercial or financial relationships that could be construed as a potential conflict of interest.

## Publisher’s note

All claims expressed in this article are solely those of the authors and do not necessarily represent those of their affiliated organizations, or those of the publisher, the editors and the reviewers. Any product that may be evaluated in this article, or claim that may be made by its manufacturer, is not guaranteed or endorsed by the publisher.

## Footnotes

^1^ https://www.agenziacoesione.gov.it/

## References

[1] Our World InData. Our world in data (2022). Available at: https://ourworldindata.org/ (Accessed December 1, 2022).

[2] Alicandro G Corsetti G Battaglini M Prati S Frova L. Education inequalities in overall mortality during the first wave of the COVID-19 pandemic in Italy. Epidemiol Prev. (2021) 45:463–9. doi: 10.19191/EP21.6.122, PMID: 35001594

[3] Mateo-Urdiales A Fabiani M Rosano A Vescio MF Del Manso M Bella A. Socioeconomic patterns and COVID-19 outcomes before, during and after the lockdown in Italy (2020). Health Place. (2021) 71:102642. doi: 10.1016/j.healthplace.2021.102642, PMID: 34339938 PMC8318679

[4] Petrelli A Di Napoli A. The impact of COVID-19 on the immigrant population in Italy. Context, methodology and synthesis of the main evidence from the project of the National Institute for Health, Migration and Poverty (INMP) and Italian Regions. Epidemiol Prev. (2022) 46:7–13. doi: 10.19191/EP22.4S1.051 PMID: 35862555

[5] Di Girolamo C Bartolini L Caranci N Moro ML. Socioeconomic inequalities in overall and COVID-19 mortality during the first outbreak peak in Emilia-Romagna region (northern Italy). Epidemiol Prev. (2020) 44:288–96. doi: 10.19191/EP20.5-6.S2.129, PMID: 33412821

[6] Petrelli A Di Napoli A Sebastiani G Rossi A Giorgi Rossi P Demuru E. Italian atlas of mortality inequalities by education level. Epidemiol Prev. (2019) 43:1–120. doi: 10.19191/EP19.1.S1.002, PMID: 30808126

[7] Istat. Indicatori demografici – anno 2021 (2022).

[8] Carnegie ER Inglis G Taylor A Bak-Klimek A Okoye O. Is population density associated with non-communicable disease in Western developed countries? A systematic review. Int J Environ Res Public Health. (2022) 19:2638. doi: 10.3390/ijerph19052638, PMID: 35270337 PMC8910328

[9] Passchier-Vermeer W Passchier WF. Noise exposure and public health. Environ Health Perspect. (2000) 108 Suppl 1:123–31. doi: 10.1289/ehp.00108s1123, PMID: 10698728 PMC1637786

[10] Castillo-Reinado K Maier W Holle R Stahl-Pehe A Baechle C Kuss O. Associations of area deprivation and urban/rural traits with the incidence of type 1 diabetes: analysis at the municipality level in North Rhine-Westphalia, Germany. Diabet Med. (2020) 37:2089–97. doi: 10.1111/dme.14258, PMID: 31999840

[11] Su B Wang Y Dong Y Hu G Xu Y Peng X. Trends in diabetes mortality in urban and rural China, 1987-2019: a Joinpoint regression analysis. Front Endocrinol (Lausanne). (2021) 12:777654. doi: 10.3389/fendo.2021.777654 35111135 PMC8801697

[12] Yu L Sabatino SA White MC. Rural-urban and racial/ethnic disparities in invasive cervical cancer incidence in the United States, 2010-2014. Prev Chronic Dis. (2019) 16:E70. doi: 10.5888/pcd16.180447 31172917 PMC6583816

[13] Zahnd WE James AS Jenkins WD Izadi SR Fogleman AJ Steward DE. Rural-urban differences in cancer incidence and trends in the United States. Cancer Epidemiol Biomark Prev. (2018) 27:1265–74. doi: 10.1158/1055-9965.EPI-17-0430, PMID: 28751476 PMC5787045

[14] Casant J Helbich M. Inequalities of suicide mortality across urban and rural areas: a literature review. Int J Environ Res Public Health. (2022) 19:19. doi: 10.3390/ijerph19052669 PMC8909802 35270369

[15] Cross SH Mehra MR Bhatt DL Nasir K O'Donnell CJ Califf RM. Rural-urban differences in cardiovascular mortality in the US, 1999-2017. JAMA. (2020) 323:1852–4. doi: 10.1001/jama.2020.2047, PMID: 32396176 PMC7218488

[16] Bremberg S. Rural-urban mortality inequalities in four Nordic welfare states. Scand J Public Health. (2020) 48:791–3. doi: 10.1177/1403494820921684, PMID: 32456534 PMC7678336

[17] Probst JC Zahnd WE Hung P Eberth JM Crouch EL Merrell MA. Rural-urban mortality disparities: variations across causes of death and race/ethnicity, 2013-2017. Am J Public Health. (2020) 110:1325–7. doi: 10.2105/AJPH.2020.305703, PMID: 32673111 PMC7427230

[18] Li X Rudolph AE Mennis J. Association between population mobility reductions and new COVID-19 diagnoses in the United States along the urban-rural gradient, February-April, 2020. Prev Chronic Dis. (2020) 17:E118. doi: 10.5888/pcd17.200241 33006542 PMC7553217

[19] Islam N Lacey B Shabnam S Erzurumluoglu AM Dambha-Miller H Chowell G. Social inequality and the syndemic of chronic disease and COVID-19: county-level analysis in the USA. J Epidemiol Community Health. (2021) 75:496–500. doi: 10.1136/jech-2020-215626, PMID: 33402397

[20] Lee J Ramírez IJ. Geography of disparity: connecting COVID-19 vulnerability and social determinants of health in Colorado. Behav Med. (2022) 48:72–84. doi: 10.1080/08964289.2021.2021382, PMID: 35318900

[21] Cuadros DF Branscum AJ Mukandavire Z Miller FD MacKinnon N. Dynamics of the COVID-19 epidemic in urban and rural areas in the United States. Ann Epidemiol. (2021) 59:16–20. doi: 10.1016/j.annepidem.2021.04.007, PMID: 33894385 PMC8061094

[22] Denslow S Wingert JR Hanchate AD Rote A Westreich D Sexton L. Rural-urban outcome differences associated with COVID-19 hospitalizations in North Carolina. PLoS One. (2022) 17:e0271755. doi: 10.1371/journal.pone.0271755, PMID: 35976813 PMC9384999

[23] Huang Q Jackson S Derakhshan S Lee L Pham E Jackson A. Urban-rural differences in COVID-19 exposures and outcomes in the south: a preliminary analysis of South Carolina. PLoS One. (2021) 16:e0246548. doi: 10.1371/journal.pone.0246548, PMID: 33534870 PMC7857563

[24] ISS Sistema di sorveglianza integrata COVID-19. (2020). Available at: https://www.epicentro.iss.it/coronavirus/sars-cov-2-sorveglianza (Accessed March 20, 2023).

[25] Chen JT Krieger N. Revealing the unequal burden of COVID-19 by income, race/ethnicity, and household crowding: US County versus zip code analyses. J Public Health Manag Pract. (2021) 27, COVID-19 and Public Health: Looking Back, Moving Forward:S43–s56. doi: 10.1097/PHH.0000000000001263 32956299

[26] Islam SJ Nayak A Hu Y Mehta A Dieppa K Almuwaqqat Z. Temporal trends in the association of social vulnerability and race/ethnicity with county-level COVID-19 incidence and outcomes in the USA: an ecological analysis. BMJ Open. (2021) 11:e048086. doi: 10.1136/bmjopen-2020-048086, PMID: 34301657 PMC8300549

[27] Zhai W Liu M Fu X Peng Z-R. American inequality meets COVID-19: uneven spread of the disease across communities. Ann Am Assoc Geogr. (2021) 111:2023–43. doi: 10.1080/24694452.2020.1866489

[28] Miguel CB da Silva AL Trindade-da-Silva CA de Abreu MCM Oliveira CJF Rodrigues WF. Proximity matrix indicates heterogeneity in the ability to face child malnutrition and pandemics in Brazil: an ecological study. Front Public Health. (2022) 10:1019300. doi: 10.3389/fpubh.2022.1019300, PMID: 36438240 PMC9686321

[29] Vallee A. Heterogeneity of the COVID-19 pandemic in the United States of America: a geo-epidemiological perspective. Front Public Health. (2022) 10:818989. doi: 10.3389/fpubh.2022.818989, PMID: 35155328 PMC8826232

[30] ISS. Sorveglianza integrata COVID-19: archivio dei principali dati nazionali (2023).

[31] ACT. Agenzia per la Coesione Territoriale (ACT). Strategia Nazionale Aree Interne. (2020) Available at: https://www.agenziacoesione.gov.it/strategia-nazionale-aree-interne/ (Accessed December 1, 2022).

[32] ISS. Monitoraggio delle varianti del virus SARS-CoV-2 di interesse in sanità pubblica in Italia. (2021) Available at: https://www.epicentro.iss.it/coronavirus/sars-cov-2-monitoraggio-varianti-indagini-rapide (Accessed February 1, 2023).

[33] Istat. L’indice di vulnerabilità sociale e materiale. (n.d.). Available at: https://ottomilacensus.istat.it/fileadmin/download/Indice_di_vulnerabilit%C3%A0_sociale_e_materiale.pdf (Accessed March 8, 2023).

[34] Agnoletti M Manganelli S Piras F. Covid-19 and rural landscape: the case of Italy. Landsc Urban Plan. (2020) 204:103955. doi: 10.1016/j.landurbplan.2020.103955, PMID: 32994653 PMC7515565

[35] CDC. COVID-19 stats: COVID-19 incidence, * by urban-rural classification(†) – United States, January 22-October 31, 2020(§). MMWR Morb Mortal Wkly Rep. (2020) 69:1753. doi: 10.15585/mmwr.mm6946a6 33211682 PMC7676636

[36] Greteman BB Garcia-Auguste CJ Gryzlak BM Kahl AR Lutgendorf SK Chrischilles EA. Rural and urban differences in perceptions, behaviors, and health care disruptions during the COVID-19 pandemic. J Rural Health. (2022) 38:932–44. doi: 10.1111/jrh.12667, PMID: 35466479 PMC9115219

[37] Saelee R Zell E Murthy BP Castro-Roman P Fast H Meng L. Disparities in COVID-19 vaccination coverage between urban and rural counties – United States, December 14, 2020-January 31, 2022. MMWR Morb Mortal Wkly Rep. (2022) 71:335–40. doi: 10.15585/mmwr.mm7109a2, PMID: 35239636 PMC8893338

[38] Sun Y Monnat SM. Rural-urban and within-rural differences in COVID-19 vaccination rates. J Rural Health. (2021) 38:916–22. doi: 10.1111/jrh.12625 34555222 PMC8661570

[39] Petrelli AM. S. La mortalità nelle "aree interne": una lettura originale della salute disuguale nel territorio italiano. (2019). Available at: https://www.slideshare.net/slideistat/apetrelli-la-moltalit-nelle-aree-interne-una-lettura-originale-della-saljute-disuguale-nel-terriroio-italkuano (Accessed December 1, 2022).

[40] Maroko AR Nash D Pavilonis BT. COVID-19 and inequity: a comparative spatial analysis of New York City and Chicago hot spots. J Urban Health. (2020) 97:461–70. doi: 10.1007/s11524-020-00468-0, PMID: 32691212 PMC7371785

[41] Bertoncello C Cazzaro R Ferraresso A Mazzer R Moretti G. Prevalence of overweight and obesity among school-aged children in urban, rural and mountain areas of the Veneto region, Italy. Public Health Nutr. (2008) 11:887–90. doi: 10.1017/S1368980007001152, PMID: 17942006

[42] Cyril SO Oldroyd JC Renzaho A. Urbanisation, urbanicity, and health: a systematic review of the reliability and validity of urbanicity scales. BMC Public Health. (2013) 13:513. doi: 10.1186/1471-2458-13-513, PMID: 23714282 PMC3671972

