# Supplementary material for: Geographic heterogeneity of the epidemiological impact of the COVID-19 pandemic in Italy using a socioeconomic proxy-based classification of the national territory
Source: Front Public Health. 2023 Apr 21;11:1143189. doi: 10.3389/fpubh.2023.1143189 (PMC10160611; doi:10.3389/fpubh.2023.1143189)
Supplement: Supplementary file 1 [file Table_1.docx]

**Supplementary Table 1. Distribution of COVID-19 cases, non-intensive and intensive care hospital admissions, and deaths. Crude and age-standardized rates, with 95% confidence intervals (CI) for all the study outcomes, by geographic area, class of municipalities and time period.**

**Supplementary Table 2.** **COVID-19 vaccination access. Crude and age-standardized rates, with 95% confidence intervals (CI) by geographic area and class of municipalities.**
